# Breakthrough and Episodic Cancer Pain from a Palliative Care Perspective

**DOI:** 10.3390/curroncol30120746

**Published:** 2023-11-30

**Authors:** Erik Torbjørn Løhre, Gunnhild Jakobsen, Tora Skeidsvoll Solheim, Pål Klepstad, Morten Thronæs

**Affiliations:** 1 Cancer Clinic, St. Olavs Hospital, Trondheim University Hospital, 7030 Trondheim, Norway; erik.t.lohre@ntnu.no (E.T.L.);; 2 Department of Clinical and Molecular Medicine, Faculty of Medicine and Health Sciences, NTNU–Norwegian University of Science and Technology, 7030 Trondheim, Norway; 3 Centre for Crisis Psychology, Faculty of Psychology, University of Bergen, 5007 Bergen, Norway; 4 Department of Public Health and Nursing, Faculty of Medicine and Health Sciences, NTNU–Norwegian University of Science and Technology, 7030 Trondheim, Norway; 5 Department of Anesthesiology and Intensive Care Medicine, St. Olavs Hospital, Trondheim University Hospital, 7030 Trondheim, Norway; 6 Department of Circulation and Medical Imaging, Faculty of Medicine and Health Sciences, NTNU–Norwegian University of Science and Technology, 7030 Trondheim, Norway

**Keywords:** palliative care, acute palliative care unit (APCU), cancer pain, breakthrough cancer pain, episodic cancer pain, pain flares, background pain

## Abstract

Cancer pain intensity (PI) fluctuates, but the relationship between pain flares and background pain with respect to pain management is not settled. We studied how flare and background PIs corresponded with treatment results for background cancer pain. Patients admitted to an acute palliative care unit with average and/or worst PI ≥ 1 on the 11-point numeric rating scale were included. Average and worst PI at admission and average PI at discharge were collected. We examined how the difference and ratio between worst and average PI and average PI at admission, were associated with average PI development during hospitalization. Positive differences between worst and average PI at admission were defined as pain flares. Ninety out of 131 patients had pain flares. The reduction in average PI for patients with flares was 0.9 and for those without, 1.9 (*p* = 0.02). Patients with large worst minus average PI differences reported the least improvement, as did those with large worst/average PI ratios. Patients with pain flares and average PI ≤ 4 at admission had unchanged average PI during hospitalization, while those with pain flares and average PI > 4 experienced pain reduction (2.1, *p* < 0.001). Large pain flares, in absolute values and compared to background PI, were associated with inferior pain relief.

## 1. Introduction

Despite the ongoing focus, pain management is an insufficiently solved problem in palliative cancer care [1]. And in fact, the situation has not improved over the past years or decades [1,2]. In addition, previous studies conducted by our group demonstrated that everyday pain management in an acute palliative care unit (APCU) was inferior to that obtained during a more controlled and standardized study context [3,4]. To reach the pain treatment goals defined by the patient or the physician, repeated evaluations of worst and average pain intensities (PIs) are crucial, as cancer pain may fluctuate and increase both due to cancer progression and treatment interventions [5,6,7,8,9,10,11,12]. Thereafter, the systematic pain assessment must be followed by the administration of therapies designed both to control sustained and chronic cancer pain and short-lived cancer pain exacerbations [13,14].

Breakthrough cancer pain (BTCP) has, since the expression emerged, been explained as an episode of high PI that “breaks through” the continuous and controlled background cancer pain [10]. In contemporary research, and in contrast to the pioneer definition, controlled background pain is defined as being of “mild” intensity [15,16]. Since the introduction of the term BTCP, background pain has been assessed as average PI [16,17]. Based on interference with patient function and a systematic review of the literature, pain intensities 1–4 on the 11-point numeric rating scale (NRS 0–10) correspond to mild pain [18,19]. On the other hand, researchers advocate different opinions on how to assess transient cancer pain exacerbations and at which intensity they can be defined as BTCP [20]. The fact that the intensity of pain flares may vary with time adds complexity to the picture [21]. Additionally, BTCP is an English term with no literal translation in many languages, and international studies have demonstrated a higher prevalence of reported BTCP in English-speaking and Northern and Western European countries [22,23].

From previous research, it can be extracted that background PI is close to normally distributed in cancer patients with intermittent pain flares [17,24]. This implies that patients with pain flares may have no background pain, controlled background pain, or uncontrolled background pain [14]. And thus, many patients with transient pain exacerbations have uncontrolled background pain. These patients have by definition no BTCP, but they still suffer from intermittent pain flares. Based on these facts, “episodic pain” was suggested as an overarching concept to generically describe all significant intermittent pain flares in cancer patients, whether their background PI is controlled or not [20]. In a review of classification in the 11th revision of the World Health Organization’s International Classification of Diseases (ICD-11), the temporal characteristics of cancer pain were described as continuous background pain and intermittent episodic pain [25].

The relationship between pain flares and background PI has been studied, and it is observed that optimization of uncontrolled background PI may have positive effects on pain flares [26]. Identifying the optimum analgetic regimen for pain flares may, however, represent a therapeutic challenge [14]. With a potential time to maximum PI of less than 10 min and the mean duration of an untreated pain flare of less than an hour, finding the appropriate medication may be demanding [27]. A predictable pain flare induced by a specific activity may be treated with an immediate-release opioid prior to the activity [14]. However, increased opioid blood concentration after the pain flare has vanished may result in unwanted side effects [14]. Transmucosal fentanyl formulations represent short-acting and potent alternatives for pain flares [14]. They are currently not recommended for patients using less than 60 mg oral morphine equivalents per day, and concerns about misuse and overdoses also may have limited the clinical use of transmucosal fentanyl formulations [13,28]. Continuous parenteral administration of opioids, whether patient-controlled or not, is commonly used in APCUs, but their mere application represents no guarantee that boluses are given when indicated for pain flares [14]. Still, transmucosal fentanyl formulations may be relevant in patients with unpredictable and rapid-onset pain flares and parenteral opioids in patients with severe cancer pain [13].

To gain more insight into the importance of the intensity of intermittent pain flares and background PI for treatment results during the hospital stay, we conducted a secondary analysis of a study of patients with cancer pain who were admitted to an APCU. The primary objective was to investigate if patients with more intense pain flares at admission achieved inferior treatment outcomes for average PI during the hospital stay. The secondary objectives were to explore whether the ratio between worst and average PI and the level of background PI at admission (controlled vs uncontrolled) were related to average PI relief during hospitalization for patients with pain flares. As an exploratory objective, we examined to which degree transmucosal fentanyl formulations and parenteral opioids were used during the hospital stay in patients with the most intense pain flares.

## 2. Materials and Methods

### 2.1. Design

Recently, results from a prospective longitudinal observational study conducted at the APCU, Cancer Clinic, St. Olavs Hospital, Trondheim University Hospital, Norway were published [4]. The Cancer Clinic has long been an ESMO designated center of integrated oncology and palliative care, and the APCU has 12 beds and approximately 450 admissions a year. In the primary observational study, all patients admitted between 15 January 2019 and 15 January 2020 were assessed. The current study is a secondary analysis of results from the primary study, which focused on the use of interventions and symptom relief during the stay [4].

### 2.2. Patients

The population referred to the APCU consists of adult patients with incurable cancer and in need of palliative care interventions. Both patients with concurrent and no ongoing cancer treatment are admitted, and most patients are admitted from their homes. Patients with hematological, gynecological, and pulmonary malignancies are treated at the respective University hospital departments, and they are only referred to the APCU when neuraxial pain management is indicated. This analysis included all patients with self-reported average and/or worst PI ≥ 1 (NRS 0–10) at hospitalization and available self-reported pain registrations both at admission and discharge. Readmissions were not included in the analyses.

### 2.3. Assessments

Patient-reported worst and average PIs (NRS 0–10) at admission and average PI at discharge were collected from the primary data files. The assessment period for all PIs was past 24 h. Reduction in average PI from admission to discharge was used as the measure for pain relief for both the primary and the secondary objectives. Furthermore, we collected physician-recorded information on patient demographics, including age, gender, cancer diagnosis, metastatic status, Eastern Cooperative Oncology Group (ECOG) performance status, care trajectory (receiving palliative care only or integrated oncology and palliative care services), medical comorbidity, and opioid medication [29].

### 2.4. Statistical Analysis

To investigate pain relief during hospitalization for patients with and without pain flares, the NRS 0–10 differences between the worst and average PI at admission were calculated for all patients. The group with positive differences was defined as having pain flares, and the group without positive differences as not having pain flares. Mean changes in average PI from admission to discharge were calculated to explore pain relief and compared for the two groups. An independent sample t-test was used for group comparison.

To study the relationship between pain flare intensity and pain relief for patients with pain flares, NRS 0–10 differences between worst and average PI at admission were calculated and defined as the absolute pain spike intensity. The absolute pain spike intensities were grouped according to the size of the differences. The applied groups for absolute pain spike intensities were NRS 0–10 differences 1, 2, 3, and ≥4, respectively. Mean changes in average PI from admission to discharge were compared for the different groups. The analyses were conducted using an ANOVA model.

To explore if the ratio between worst and average PI had an impact on pain relief for patients with pain flares, NRS 0–10 differences between worst and average PI divided by average PI at admission were calculated. For the current purpose, this fraction was defined as the relative pain spike intensity. The relative pain spike intensities were grouped according to the size of the fractions. The applied intervals for relative pain spike intensities were <0.49, 0.5–0.99, 1.0–1.49, and ≥1.5. Mean changes in average PI from admission to discharge were compared for the different groups. The analyses were conducted using an ANOVA model.

To estimate whether level of background PI was associated with pain relief during hospitalization in patients with pain flares, the patients were divided into two groups. The groups consisted of patients with average PI ≤ 4 (controlled background pain) and patients with average PI > 4 (uncontrolled background pain). Mean changes in average PI from admission to discharge were compared for the two groups. An independent sample t-test was used for group comparison.

To examine to which degree transmucosal fentanyl formulations and parenteral opioids were used by patients with the most intense flares during the hospital stay, the patients were divided into three groups. The first group consisted of patients with absolute pain spike intensity ≥ 4, the second included the remaining group of patients with pain flares, and the third group consisted of patients with no pain flares at admission. The three groups were compared, with respect to the use of transmucosal fentanyl formulations, parenteral opioids, and peroral opioids, using Pearson’s chi-squared test.

Equal variances were checked for using Levene’s test, and normal distributions were verified by visual inspection of P-P plots. *p*-values ≤ 0.05 were considered significant. The statistical analyses were performed using SPSS statistical software (Version 27.0) (International Business Machines (IBM), Armonk, NY, USA).

### 2.5. Ethics

The Regional Committee for Medical Research Ethics, Health Region Central Norway (REK) (2018/925/REK midt and 2021/212312/REK midt) defined the primary project and secondary analyses as healthcare improvement, without the need for explicit informed consent from the patients.

## 3. Results

### 3.1. Demographics

Out of the 451 admissions included in the primary study, 195 readmissions were excluded. One hundred thirty-one unique patients had average and/or worst PI of at least 1 (NRS 0–10) at admission, reported worst and average PI at admission, and average PI at discharge. These 131 patients were included in the current analysis. The baseline patient characteristics are described in Table 1. The mean age was 70 years, and 67% were males. Gastrointestinal, urological, and breast cancer were the most frequent cancer diagnoses, and all had metastatic disease. Median ECOG status was III (range I–IV), and 44% received integrated oncology and palliative care services. Strong opioids were prescribed for 117 patients and administered as a continuous parenteral infusion in 48 patients.

### 3.2. Pain Alleviation during Hospitalization for Patients with and without Pain Flares

Ninety patients (69%) had a positive NRS 0–10 difference between worst and average PI at admission and were defined as having pain flares, and 41 (31%) had no pain flares. Mean reduction in average PI during hospitalization for patients with flares was 0.9, and for patients without flares 1.9 (*p* = 0.02).

### 3.3. Absolute Pain Spike Intensity and Pain Relief

At admission, 30 patients had an NRS difference of 1 between worst and average PI, and thus, absolute pain spike intensity of 1. Correspondingly, 17 had 2, 17 had 3, and 26 patients had absolute pain spikes that were at least 4 above the average PI. Patients with high pain spikes at admission reported less mean average PI reduction during hospitalization compared to patients with low pain spikes, and the group differences were statistically significant (*p* = 0.03). The groups of absolute pain spike intensities and corresponding mean reductions in average PI during hospitalization are presented in Table 2.

### 3.4. Relative Pain Spike Intensity and Pain Relief

At admission, 37 patients had a difference between worst and average PI divided by average PI < 0.49, and thus relative pain spike intensity < 0.49. Correspondingly, 17 had 0.5–0.99, 14 had 1.0–1.49, and 14 patients had pain spikes that were at least 1.5 times higher than the average PI. Due to a denominator of zero, eight patients with pain flares and self-reported average PI of 0 (NRS 0–10) were not applicable for these calculations. Patients with large ratios between pain spike intensity and average PI reported less mean average PI reduction during hospitalization compared to patients with small ratios. In fact, patients with pain spikes at least 1.5 higher than average PI reported worsening. The group differences were statistically significant (*p* < 0.001). The groups of relative pain spike intensities and corresponding mean changes in average PI during hospitalization are delineated in Table 3.

### 3.5. The Significance of Background Pain Intensity for Pain Control

At admission, 50 (56%) of the patients with pain flares had controlled background pain (average PI ≤ 4) and 40 (44%) with pain flares had uncontrolled background pain (average PI > 4). The patients with controlled background pain reported a mean average PI worsening of 0.1 during hospitalization, and those with uncontrolled background pain had a mean average PI reduction of 2.1 (NRS 0–10). The group differences were significant (*p* < 0.001).

### 3.6. Transmucosal Fentanyl Formulations and Parenteral Opioids in Patients with Pain Flares

None of the patients with the most intense pain flares at admission used transmucosal fentanyl formulations during the hospital stay (Table 4). Also, during the hospital stay, the percentage of patients on parenteral opioids was highest among those with the most intense pain flares. However, compared to the remaining patients with pain flares and the patients with no pain flares, the group differences were not statistically significant (*p* = 0.59). Correspondingly, the percentage of patients on peroral opioids was lowest among those with the most intense pain flares. However, compared to the remaining patients with pain flares and the patients with no pain flares, the group differences were not statistically significant (*p* = 0.34). Among the patients with the most intense pain flares at admission, similar percentages used parenteral and peroral opioids during the hospital stay (*p* = 0.32).

## 4. Discussion

### 4.1. Statement of Principal Findings

We demonstrated that palliative cancer care patients with high pain spikes at admission experienced less improvement of average PI during hospitalization compared to patients with low pain spikes or no pain spikes. Those with the largest spike/average PI ratios even reported pain worsening during the hospital stay. Almost one-third of the patients had pain flares and uncontrolled background pain. This information is relevant as patients with pain flares and uncontrolled background pain reported better pain relief compared to those with pain flares and controlled background pain.

### 4.2. Appraisal of Methods

Retrospective analyses have important design limitations [30]. They are prone to both selection and recall biases and are unable to establish cause–effect relationships. Hence, overgeneralization of the results should be avoided. However, the results may generate hypotheses for future prospective studies [30]. The present study is a secondary analysis of data from a study originally designed to describe interventions and symptom relief in an APCU [4]. Thus, the analyzed data were not collected to address the study objectives [31]. In addition, even though designated BTCP assessment tools exist, none of these were applied in the current study [6,32]. Furthermore, no evaluations of pain mechanisms, psychological distress, addictive behavior, or cognitive function were included [33]. Moreover, the occurrence of pain flares was estimated only at admission, which at best only provides a clinically relevant snapshot of a complex pain situation possibly changing throughout the hospital stay. Given the study objectives, one might also argue that evaluations of the worst PI development during hospitalization would be relevant. However, more details and complexity might have come at the cost of clarity. Still, we present a large dataset, where patient-reported PIs were obtained prospectively with recommended and previously applied methodology [3,34,35].

### 4.3. Comparison with Previous Work

Temporal variations in cancer PI are often difficult to manage, and baseline PI may be both high and low in patients with pain flares [6,17]. An observational cross-sectional multicenter multinational study that also included patients with uncontrolled background pain demonstrated that patients with pain flares had higher average PI than those without [36,37]. This finding was in line with the results from a previous international survey demonstrating that average PI was higher in patients with BTCP compared to patients without [23]. The observations are both consistent with our results showing that patients with pain flares past 24 h reported less pain relief during hospitalization compared to those with no pain flares past 24 h at admission.

Patient-reported PI is regarded as the standard for pain assessment and the first necessary step towards effective and individualized pain treatment [13,38]. In early research, transient episodes of severe or excruciating PI were used as rule-in criteria for BTCP [16,39]. More recently, BTCP intensities are described as both moderate and severe, and for some patients, even as mild [40]. Whereas early research considered moderate background PI controlled, later BTCP definitions allowed for no higher than mild background PI [15,16]. With the introduction of a numeric cut-off for controlled background pain, the scene was set for even more precise descriptions of both peak and background PIs [26,27,41]. Hence, associations between peak and background PI were disclosed, and optimized pain control was shown to decrease both the number, intensity, and duration of BTCP episodes [26,27]. In the current paper, clinical implications of high absolute pain spike intensity were further explored, and we demonstrated that patients with large differences between worst and average PI achieved inferior pain treatment results.

Large worst/average PI ratios are indicative of considerable cancer pain fluctuations. Cancer pain may be related to skeletal metastases, visceral metastases, and cancer-related neuropathic pain [25]. These disease manifestations may cause considerable cancer pain fluctuations and a call for treatment interventions other than opioids [42,43,44]. For selected patients, single-fraction external beam radiotherapy or neurolytic blocks may be relevant and necessary, and for some patients, the addition of an antidepressant or anticonvulsive may suffice [13]. All patients included in the current study had metastases, and 42% had skeletal metastases. Since no other information on pain mechanisms was available, one may only speculate if suboptimal therapy choices prior to admission contributed to inferior pain relief in patients with high relative pain spike intensity [38].

Clinical studies have demonstrated the fact, and experts have acknowledged, that patients with uncontrolled background pain may experience pain flares [17,20,24]. In the current study, we observed that almost one-third of the patients had pain flares and uncontrolled background pain. The percentage is a little lower than the number of patients with pain flares and controlled background pain and somewhat higher than the percentage of patients with pain flares and uncontrolled background pain described in previous research [17,24]. Still, the condition is relevant for many patients, and pain flares outside the definition of BTCP must be accounted for in research and addressed in clinical practice [20,24].

Due to the rapid onset and short duration of increased PI, BTCP is considered difficult to manage [45,46]. These previous findings are in line with our current observations, indicating that patients with pain flares and controlled background pain experienced no pain relief during hospitalization in an APCU with a dedicated focus on symptom management. This group may include patients with controlled background pain and little tolerance for the increased opioid doses needed to control pain flares. Perhaps patients with uncontrolled background pain, in general, are more likely to tolerate increased opioid doses resulting in more controlled background pain and fewer pain flares. Contrary to this, patients with pain flares but no background pain may be more difficult to treat sufficiently, as their flares may have subsided before the opioid is metabolized, with relative overdosing as a consequence [14]. The primary study, which the current results were drawn from, also demonstrated that patients with high PI at admission reported pain relief superior to those with low [4]. One possible interpretation is that the potential for further improvement is smaller on the lower end of the NRS scale. Another plausible inference is that patients with high average PI at admission have a potential for improvement that should be utilized in clinical practice.

Transmucosal fentanyl formulations and parenteral opioids may be indicated in patients with difficult-to-treat cancer pain [13,14]. However, finding the drug, formulation, and dosing resulting in treatment responses mimicking rapid waxing and waning PIs can represent a treatment challenge, especially considering opioids with potentially extensive side effects [14]. Still, optimizing cancer pain management is important, and recently a more personalized approach has been called for [1,47]. This might include individually adapted alternatives for both drugs, formulations, and dosings. Our exploratory results provide limited guidance on the topic. Still, the fact that patients with the most intense pain flares did not use rapid onset opioid formulations to an even greater extent, might perhaps support that both specific assessment of pain flare intensities and a personalized therapeutic approach may represent steps towards improved cancer pain management.

### 4.4. Clinical and Scientific Implications

Rapid-onset and short-lived cancer pain exacerbations of high intensity represent a therapeutic challenge in patients both with controlled and uncontrolled background pain. Given the heterogeneity and complexity of cancer pain, a thorough diagnostic work-up, including patient reports, anamnesis, physical examination, labs, and imaging, may be needed to disclose the underlying pathophysiological pain mechanisms [20,38]. Our results indicate that evaluation of the magnitude of pain flares might be relevant to the diagnostic process. Diagnosing somatic and visceral nociceptive pain and neuropathic cancer pain may be crucial for choosing the optimal medical, radiological, and interventional treatment options [13,14]. In addition, the prompt availability of short-acting opioids is needed [14]. For many patients, transmucosal fentanyl may represent the best treatment option for breakthrough and episodic cancer pain [14,48]. For others, oral immediate release opioids may be sufficient, and for some patients, parenteral or intrathecal opioids necessary for pain control [14].

The present study supports that assessment of transient cancer pain exacerbations is relevant for all patients and not only for those with background PI below a certain cut-off level. Estimates on the intensity of the pain flares and their relation to background PI represent clinically relevant pain descriptors applicable in science.

Episodic cancer pain describes pain flares in patients with both controlled and uncontrolled background cancer pain. By applying this umbrella term, one acknowledges the presence of pain flares in patients with uncontrolled background pain and adds precision to the expression BTCP.

### 4.5. Further Work

With the inherent limitations of the current data set, future prospective studies designed to address pain flares are needed to confirm or reject the findings. Further studies on the clinical relevance of pain flares in patients with uncontrolled background pain are probably warranted, and one may hypothesize that more exhaustive descriptions of transient cancer pain exacerbations could improve BTCP research.

## 5. Conclusions

Based on interpretations of retrospective real-life data, high pain spikes and large variations in PI were indicative of inferior pain management outcomes. Both patients with and without controlled background pain experienced pain flares, but those with controlled background pain had the poorest treatment outcomes.

## Figures and Tables

**Table 1:** Patient demographics (n = 131).

Characteristics	Sample	Percentage
Gender, n (%)		
Female	43	32.8
Male	88	67.2
Age, years, mean (SD ^1^)	70 (13)	
Cancer diagnosis, n (%)		
Gastrointestinal	59	45.0
Urological	33	25.2
Breast	10	7.6
Lung	2	1.5
Head/Neck	8	6.1
Others	18	13.7
Missing Metastatic status, n (%)	1	0.8
Metastases present	131	100
Skeletal metastases	55	42.0
Comorbidity ^2^, n (%)		
Yes	95	72.5
No	36	27.5
WHO performance status, n (%)		
WHO 1–2	64	48.9
WHO 3–4	67	51.1
Care trajectory, n (%)		
Palliative care	72	55.0
Integrated oncology and palliative care	58	44.3
Missing	1	0.8
Pain flares, n (%)		
Pain flares present	90	68.7
No pain flares	41	31.3

^1^ SD = Standard deviation, ^2^ cardiovascular disease, diabetes, renal failure, musculoskeletal disease, psychological illness, chronic obstructive pulmonary disease, liver disease, and others.

**Table 2:** Absolute pain spike intensity and mean reduction in average pain intensity during hospitalization (n = 131).

Absolute Peak Size	n	Mean	SD ^1^
No pain spikes	41	1.93	2.3
Absolute pain spike intensity 1	30	1.43	2.5
Absolute pain spike intensity 2	17	1.18	2.5
Absolute pain spike intensity 3	17	0.94	2.3
Absolute pain spikes intensity ≥4	26	0.08	1.9

^1^ SD = Standard deviation.

**Table 3:** Relative pain spike intensity and mean reduction in average pain intensity during hospitalization (n = 82).

Peak Ratio Group	n	Mean	SD ^1^
Relative pain spike intensity <0.49	37	1.92	2.6
Relative pain spike intensity 0.5–0.99	17	1.24	1.5
Relative pain spike intensity 1.0–1.49	14	0.00	1.8
Relative pain spike intensity ≥1.5	14	−0.71 ^2^	2.1

^1^ SD = Standard deviation, ^2^ The negative number indicates average pain intensity worsening during hospitalization.

**Table 4:** Transmucosal fentanyl and parenteral opioids during the hospital stay ^1^.

Group	n	Transmucosal Fentanyl, n (%)	Parenteral Opioids, n (%)	Peroral Opioids, n (%)
Patients with absolute pain spike intensity ≥4	26	0	12 (46.2%)	11 (42.3%)
The remaining group of patients with pain flares	64	2 (3.1%)	23 (35.9%)	38 (59.4%)
Patients with no pain flares	41	1 (2.4%)	14 (34.1%)	22 (53.7%)

^1^ The table is not exhaustive on pain management, and the percentages do not add up to 100%.

## Data Availability

The datasets generated during and/or analyzed during the current study are available from the corresponding author upon reasonable request.
